# Seismic and experimental insights into eruption precursors at Volcán de Colima

**DOI:** 10.1002/2017GL073350

**Published:** 2017-06-28

**Authors:** Oliver D. Lamb, Silvio De Angelis, Richard J. Wall, Anthony Lamur, Nick R. Varley, Gabriel Reyes‐Dávila, Raúl Arámbula‐Mendoza, Adrian J. Hornby, Jackie E. Kendrick, Yan Lavallée

**Affiliations:** ^1^ Department of Earth, Ocean and Ecological Sciences University of Liverpool Liverpool UK; ^2^ Facultad de Ciencias Universidad de Colima Colima Mexico; ^3^ Centro Universitario de Estudios e Investigaciones Vulcanología Universidad de Colima Colima Mexico

**Keywords:** volcano, seismic, experimental, velocity change

## Abstract

We combine geophysical and experimental observations to interpret preeruptive unrest at Volcán de Colima in 1998. 17,893 volcanic earthquakes were detected between 1 October and 31 December 1998, including 504 clusters. Using seismic ambient noise interferometry, we observe a drop in velocity prior to the eruption linked to damage accumulation during magma ascent. This is supported by experimental observations where static stress causes a velocity decrease prior to failure. Furthermore, we observe acoustic emission clusters during the experiments, with lower porosity samples producing higher numbers of repeaters. This behavior introduces tensile failure as an additional viable mechanism for clusters during magma ascent. The findings suggest that preeruptive magma ascent may be monitored to variable degrees of accuracy via descriptions of damage accumulation and associated seismic velocity changes.

## Key Points

- Multidisciplinary seismic and experimental investigation of the 1998 eruption of Volcán de Colima
- Relative velocity decrease seen in seismic ambient noise and during experimental Brazil tests prior to eruption/failure
- Evidence for tensile failure in the volcanic edifice as a source of repeating earthquakes during magma ascent

## 1 Introduction

An important aspect of volcano monitoring is assessing whether a period of unrest will portend an eruption. Recent studies have shown that seismic interferometry, using either the coda waves of repeating earthquakes or ambient noise, holds considerable potential as a tool for monitoring active volcanoes [e.g., *Brenguier et al.*, 2008; *Hotovec‐Ellis et al.*, 2015]. Seismic wave velocities are dependent on the physical properties of the material through which they travel, and velocity changes may be induced by property changes. Seismic velocity is frequently observed to decrease prior to eruption and subsequently increase as the eruption ensues, a pattern often attributed to cycles of static stress due to magma movement [e.g., *Ratdomopurbo and Poupinet*, 1995; *Wegler et al.*, 2006; *Brenguier et al.*, 2008; *Duputel et al.*, 2009; *Hotovec‐Ellis et al.*, 2015].

Laboratory experiments have enabled relative velocity changes to be recorded over a range of pressure and temperature conditions. The accumulation of fracture damage during loading under deviatoric stresses causes an elastic velocity decrease in the tested samples [e.g., *Heap et al.*, 2010]. During rock failure tests, individual microcracking events are recorded via acoustic emissions (AEs). AEs have been demonstrated to precede material failure under temperature and stress conditions typical of shallow volcanic conduits [e.g., *Lavallée et al.*, 2008]. Further investigations have used the characteristics of AEs under controlled conditions to decipher the source mechanisms of volcanic earthquakes [e.g., *Benson et al.*, 2008; *Smith et al.*, 2009]. The recent advances in experimental rock mechanics have improved our understanding of field‐scale volcanic processes.

Very few investigations on seismic velocity changes at volcanoes have sought to replicate their results in laboratory settings. In this paper we conduct a joint seismic and experimental investigation of the mechanical response of edifice rocks prior to the November 1998 effusive eruption of Volcán de Colima, Mexico. We utilize the ambient seismic noise method on seismic data collected during this period and replicate our observations via experimental tests on samples collected from Volcán de Colima. This dual approach enables us to build a more robust interpretation of the processes occurring during preeruptive magma ascent. Ultimately, we aim to demonstrate the potential of cross‐disciplinary investigations between the fields of seismology, experimental volcanology, and rock mechanics.

### 1.1 The 1998 Eruption of Volcán De Colima

**Figure 1:**
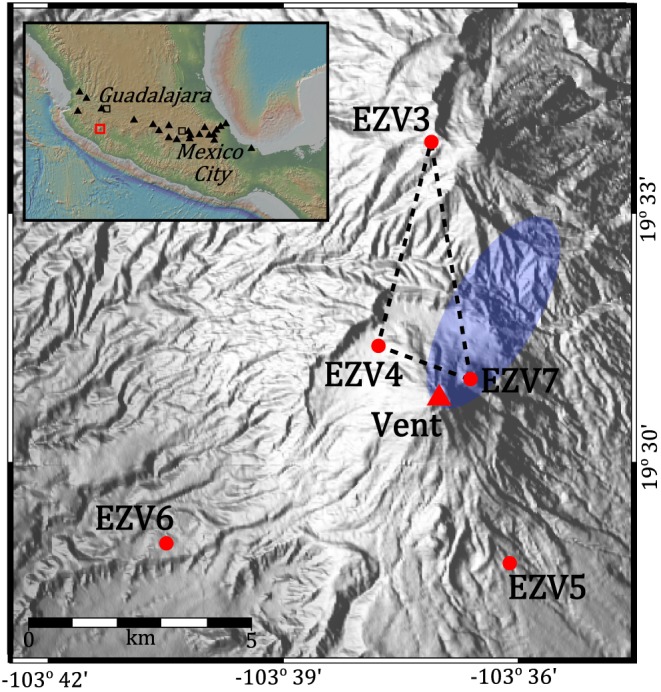
Map of VdC with the RESCO network of short‐period stations marked as they were located in 1998. The blue ellipse marks where epicenters of the November 1998 swarm were located by *Zobin et al.* [2002]. Dashed lines mark planes along which amplitude ratios and ambient noise analysis were calculated. Insert: Map showing location of VdC in Mexico (red square), relative to the major cities of Mexico City and Guadalajara (black squares). Also indicated are the locations of other Holocene volcanoes in central Mexico (triangles), as identified by the Smithsonian Global Volcanism Program [*Venzke*, 2013].

Volcán de Colima (VdC) is an andesitic stratovolcano located in western Mexico (Figure 1). From 1998 to 2011, VdC experienced multiple phases of lava extrusion, interspersed by explosive activity accompanied by emplacement of pyroclastic flows and occasional dome collapses, providing a valuable opportunity to study a rapidly evolving volcanic system. The first phase of activity was preceded by five swarms of deep and shallow volcanic earthquakes over the course of a year [*Zobin et al.*, 2002]. The final swarm in November 1998, located <3 km beneath the summit, heralded the appearance of the dome at the summit vent on 20 November [*Zobin et al.*, 2002]. The dome rapidly filled the summit crater before spilling over the SW rim to form a lava flow. Rockfalls and multiple pyroclastic flows originated from the repeated collapse of the flow front. This phase of extrusion ended in February 1999 with a lava flow extending to a final length of 3.8 km.

## 2 Data and Methods

### 2.1 Seismic

Seismic data used in this study were collected by the Colima Telemetric Seismic Network (Red Sísmica de Colima, RESCO), managed by Centro Universitario de Estudios e Investigaciones Vulcanología (CUEIV). In late 1998, this network consisted of five short‐period seismometers (EZV3–EZV7) located within 7 km of the volcano summit (Figure 1). Signals were telemetered to CUIEV and were recorded with a sampling rate of 100 Hz. We applied multistation earthquake detection from 1 October to 31 December 1998. Candidate trigger times were extracted from multiple stations using a short‐term average/long‐term average (STA/LTA) algorithm, on condition that an event was detected at three or more stations, assuming a reference seismic velocity of 2.5 km s^−1^ [*Núñez‐Cornú et al.*, 2010]. Seismic data were preprocessed with a bandpass filter between 1 and 7 Hz to improve signal‐to‐noise ratio.

Epicenters of 600 earthquakes prior to the eruption of VdC in 1998 were located generally NE of the summit vent [*Zobin et al.*, 2002, Figure 1]. The number and distribution of stations around VdC, as well as the emergent nature of most detected seismicity, does not enable accurate locations of more earthquakes. In order to track relative changes in the location of all seismicity prior to eruption at a higher temporal resolution, we employed the Seismic Amplitude Ratio Analysis (SARA) method [*Caudron et al.*, 2015]. This technique calculates the ratio of seismic intensity recorded at different seismic stations which are independent of seismic energy at the source. Since exceptional changes in attenuation are unlikely to occur at days‐months timescales, temporal variations in the ratios must be explained by changes in source locations. Following a similar methodology to *Caudron et al.* [2015], we calculated a 10 min average Real‐time Seismic Amplitudes (RSAM) for data from EZV3, EZV4, and EZV7 before using a 6 h rolling median filter to smooth the data. Data from EZV5 and EZV6 were not used as they were contaminated by high levels of anthropogenic noise. *Caudron et al.* [2015] noted that site effects, gains, and sensitivity changes would correspond to a vertical shift in ratios, whereas changes in attenuation and wave regime would result in dilation or contraction. Therefore, we can use the relative changes rather than actual values that would require corrections (e.g., site effects).

Clusters, or groups of earthquakes with similar waveforms, are significant as they represent nondestructive sources at, generally, a fixed location [*Iverson et al.*, 2006; *Neuberg et al.*, 2006; *Waite et al.*, 2008; *Varley et al.*, 2010; *Kendrick et al.*, 2014; *Lamb et al.*, 2015]. We built a catalogue of clusters at VdC by applying waveform cross correlation to our earthquake database. For each detected event, we use the first 5 s of the waveform; this time is sufficient as it includes the largest amplitudes of most waveforms while minimizing the contribution of background noise. Seismograms from station EZV7 were used to build the catalogue, as this station was closest to the summit vent (Figure 1) and typically has the highest signal‐to‐noise ratio. All waveforms were filtered with a 0.5–20 Hz bandpass Butterworth filter to further increase the signal‐to‐noise ratio. The normalized cross‐correlation coefficient (CCC) lies between 0 and 1, where 0 is unalike and 1 is identical, and was evaluated for every possible pair of earthquakes. A minimum CCC of 0.8 was used to define two or more earthquakes as a cluster. Using a CCC of 0.7 designates a higher proportion of earthquakes into clusters, but this is rejected as many of the clusters did not correlate visually (Figure S2 in the supporting information).

Several studies have successfully used seismic ambient noise to detect small variations in seismic velocity prior to volcanic eruptions [e.g., *Brenguier et al.*, 2008]. The method uses repeated cross correlations of seismic noise recorded at two seismic stations to assess the velocity properties of the subsurface medium between them [*Sens‐Schönfelder and Wegler*, 2006]. A key advantage of this technique is that it bypasses the need for repeating earthquakes to assess the seismic velocity [e.g., *Poupinet et al.*, 1984]. For our calculations we used the MSNoise software, an open source python package for monitoring seismic velocity changes using ambient seismic noise [*Lecocq et al.*, 2014]. A cross‐correlation function (CCF) was calculated from ambient noise seismic data from each individual pair of stations. Velocity variations were then calculated from different arrivals between individual CCFs and the reference CCF. Further details of the method and MSNoise program can be found in *Lecocq et al.* [2014]. For our calculations, instrument responses were not removed because they were identical and constant in time at all stations. CCFs were calculated for time lags of ±120 s within the frequency interval 0.5–1.1 Hz. This interval minimizes the influence of volcanic earthquakes whose peak frequencies generally ranged from 1 to 10 Hz. We used a reference function stacked from noise recorded during October 1998 and calculated changes in velocity from stacks calculated over 5 day and 10 day moving windows. A full list of the parameters used in the MSNoise package can be found in Table S1.

### 2.2 Experimental

In order to constrain the interpretation of seismological observations, we conducted deformation experiments to simulate the stressing conditions under which velocity change may be induced. We employed the Brazil test method to induce tensile failure [e.g., *Li and Wong*, 2013] in andesite lavas collected from the edifice of VdC. The tests involve the diametric compression of a sample disc (2:1 diameter:length aspect ratio), which induces tensional stresses in the orthogonal direction until the disc fails. This pseudotensile regime replicates and can be used as a proxy for the stress distribution associated with magma ascent prior to eruption. For our experiments, cylindrical samples of diameter 40 mm were drilled and then cut to a length of 20 mm, with the porosity of each disc measured using an Accupyc 1340 He pycnometer.

The Brazil tests were carried out in a 100 kN Instron uniaxial press at room temperature and compressive deformation rate of 0.4 μm s^−1^, corresponding to a diametral strain rate of 10^−5^ s^−1^ (Figure S4). During the experiments, load was recorded by an Instron Dynacell 2527 load cell at 100 Hz, and tensile stress (*σ*_*t*_) was calculated in real time. Simultaneously, two ceramic piezoelectric transducers (PZT) attached to diametrically opposing faces of the disc (Figure S4) monitored AE data at a sampling rate of 1 MHz. The signals from each transducer were first fed through 20 dB amplifiers before reaching a PAC PCI‐2 two‐channel recording system, with a bandwidth of 0.001–3 MHz, and capable of simultaneous hit‐based collection and waveform streaming. For each experiment we tracked the hits per second and the energy of each hit; energy was calculated using the root‐mean‐square of the recorded waveform. AE clusters were identified using the a similar approach to that used to identify seismic clusters (previous section); but no bandpass filter was used. To calculate elastic velocity properties of the sample, one PZT was set to produce “pulses” for the entire experiment duration while the other PZT recorded the pulses after they traveled through the sample. During the experiment, bursts of five pulses, each spaced 0.5 s apart, were triggered every 5 s. Each received pulse “burst” was stacked to increase the signal‐to‐noise ratio before using coda wave interferometry (CWI) to calculate change in velocity during the experiment. CWI uses the degree of correlation of the coda waves for two waves from different time intervals to calculate the variance in travel time perturbation. In particular, the correlation coefficient, *R*, is related to the variance of the travel time perturbation, *σ*_*τ*_, and to the frequency, ${\bar{\omega }}^{2}$, according to the following relationship [*Snieder et al.*, 2002]:

(1) $$R=1-\frac{1}{2}{\bar{\omega }}^{2}\sigma_{\tau }^{2}$$

The frequency, ${\bar{\omega }}^{2}$, can be calculated from the seismogram data, *u*(*t*):

(2) $${\bar{\omega }}^{2}=\frac{\overset{t+T}{\underset{t-T}{\int }}{\dot{u}}^{2}(t^{\prime })dt^{\prime }}{\overset{t+T}{\underset{t-T}{\int }}u^{2}(t^{\prime })dt^{\prime }}$$

where the integral is performed over a window of length 2*T* centered at time *t*. The velocity change follows from the travel time perturbation:

(3) $$\frac{\mathrm{\delta v}}{v}=-\sigma_{\tau }^{2}$$

For our reference waveform, we used the stacked waveform from the first burst. *δ* *v*/*v* was then calculated against this reference at the time of each of the following burst in the experiment. *Grêt et al.*[2006] used a similar method to track changes in rock properties in response to changes in stress, temperature, and water saturation.

## 3 Results

**Figure 2:**
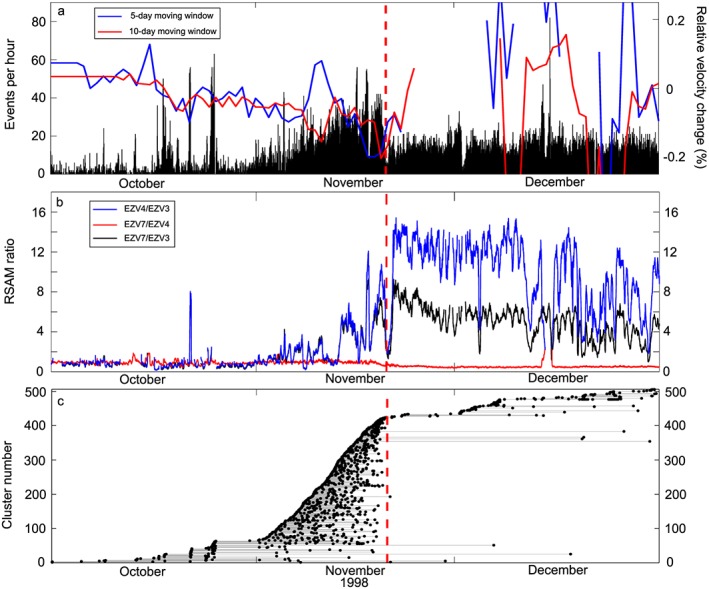
(a) Events per hour from 1 October to 31 December 1998 (black bars). Also plotted are the variations in seismic velocity calculated from 5 day (blue line) and 10 day (red) moving windows using seismic ambient noise. Gaps in lines represent periods where low coherence and large errors prevented viable calculations. The right *y* ‐axis has been limited to emphasize the change in velocity before the eruption. The full plot is available in supporting information Figure S1c. (b) Smoothed intensity ratios calculated from 10 min RSAM for EZV3, EZV4, and EZV7 stations. The original RSAM values are plotted in Figure S1. (c) Catalogue of clusters (black dots and grey lines) in our data set from 1 October to 31 December 1998. Each plotted point represents an individual earthquake, and each line joins a cluster. The red dashed line in all plots represents the beginning of the effusive eruption at VdC on 20 November 1998.

From 1 October to 31 December 1998, a total of 17,893 earthquakes were detected using our multistation detection method on data from the RESCO network (Figure 2a). We observe a gradual and steady rise in the number of seismic events detected per hour in the period 1–20 November 1998. Simultaneously, we observe an unsteady rise in the amplitude ratios of EZV4/EZV3 and EZV7/EZV3 that indicates the relative movement of earthquake sources toward stations EZV4 and EZV7 prior to the eruption on 20 November (Figure 2b). We also observe that amplitude ratios for EZV4 are higher than that of EZV7, even though the latter is closer to the summit vent (Figure 1). After 20 November, the seismic record is dominated by rockfalls originating from the lava flow on the western flank of VdC, thus explaining the larger EZV4/EZV3 ratio in the second half of our period of analysis. A total of 1313 earthquakes were defined as repeating events, representing 7.3% of the catalogue, and spread across 504 clusters containing between 2 and 22 events (Figure 2c). Three hundred fifty‐five clusters were active during the 1–20 November earthquake swarm, of which <10 continue after this period. Using the ambient seismic noise during this period, we observe a ∼0.2% decrease in seismic velocity from mid‐October to 20 November (Figure 2a). This is followed by what appears to be a sharp recovery of 0.2% shortly after the beginning of the eruption, but low coherence and large errors in measurements prevent consistent calculations during the second half of our period of analysis (Figure S3).

**Figure 3:**
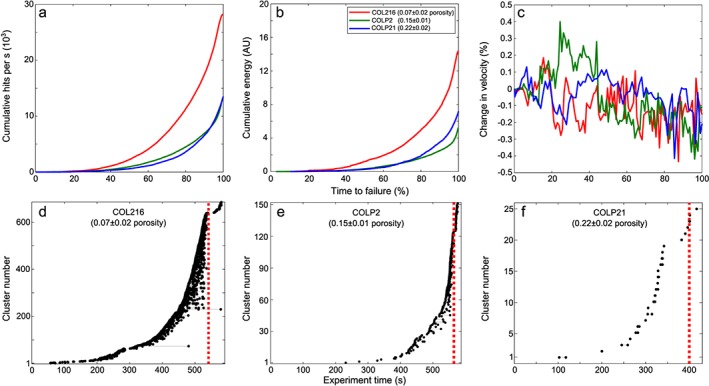
Example results for a Brazil test on discs from COL216, COLP2, and COLP21 samples. (a) Cumulative number of AEs recorded per second during experiments. (b) Cumulative energy of the recorded AE. (c) The change in velocity recorded during the experiment. (Results from all experiments are plotted in Figure S5.) Catalogues of AE clusters recorded during a Brazil test for (d) COL216 , (e) COLP2, and (f) COLP21 samples. Each black plotted point represents an individual AE, and each grey line joins groups of repeating events (i.e., clusters). The dashed red line marks the time at which the sample failed.

Ten Brazil tests were conducted on samples with a porosity constrained at 0.07 ± 0.02 (COL216), 0.15 ± 0.01 (COLP2), and 0.22 ± 0.02 (COLP21). The tensile strength of the samples was inversely proportional to the porosity, ranging between 7 and 22 MPa (Figure S5a). AE hit rate and cumulative AE energy showed an exponential increase prior to sample failure (Figures 3a and 3b). Calculated relative velocity changes during the experiments showed a consistent decrease in measurements through all samples, with no apparent relation to sample porosity (Figure 3c). We see very similar results when repeating these calculations but using the last pulse as a reference instead (Figure S6). We successfully detected AE clusters by cross‐correlating waveforms collected during one test from each sample (Figures 3d–3f). The proportion of AE clusters was higher in samples of lower porosity. For COL216, 685 groups of repeating events were detected, containing 1657 hits (5.2% of all AEs detected), COLP2 had 152 groups with 337 hits (2.3%), and COLP21 had 25 groups with 56 hits (0.37%).

## 4 Discussion

By conducting a joint seismic and experimental investigation of the 1998 eruption of VdC, we aimed to establish the potential for using multidisciplinary approaches to understand preeruptive seismic activity. The transport and eventual eruption of magma require the formation of a pathway, and in the process, rocks fracture seismogenically, providing a key proxy to forecast the eruption onset [*Smith et al.*, 2009]. Indeed, this was inferred by previous analysis of the preeruptive volcano‐seismic swarms of 1997–98 [*Zobin et al.*, 2002] and is also clearly seen in the seismic observations here (Figure 2a). Using SARA on seismic data from EZV3, EZV4, and EZV7 stations, we found that the sources of the seismic activity tended to drift toward the stations closest to the vent during this period (Figure 2b). Amplitude ratios at EZV4 tended to be higher than that of EZV7, even though the latter was located closer to the summit vent. This is likely an effect of the pyroclastic deposits where EZV4 is located, compared to the lava dome directly beneath EZV7.

Relative velocity changes measured over this period indicate a decrease of ∼0.2% prior to eruption. Low coherence and large errors in calculation prevent velocity calculations after the eruption from being well resolved. However, repeating the calculations with reference waveforms stacked from the whole period or December alone consistently calculates a slow velocity recovery following the eruption (Figures S1b and S1d). This cycle of decrease‐increase around volcanic eruptions is similar in magnitude to those seen at other volcanoes [e.g., *Ratdomopurbo and Poupinet*, 1995; *Wegler et al.*, 2006; *Brenguier et al.*, 2008; *Duputel et al.*, 2009; *Hotovec‐Ellis et al.*, 2015]. However, it does not agree with previous observations from VdC using a similar methodology [*Lesage et al.*, 2014]. Their measurements of seismic velocity from ambient noise covered a much larger period of 1998 to 2013 and observed no marked changes in relation to eruptive activity, including 1998. However, their reference cross‐correlation function was stacked from over the whole period. While this step would result in a cleaner reference function, it would also dampen the effect of eruptive activity on velocity properties. In addition, *Lesage et al.* [2014] used a broader frequency range of 0.125–2 Hz as opposed to the 0.5–1.1 Hz used here. Repeating our measurements using a reference waveform stacked from the whole period of study produces dampened velocity change decrease prior to the eruption (Figure S1b). Furthermore, using the wider‐frequency range produces erratic measurements that are consistent with our assertion that the volcanic earthquakes would influence the velocity change calculations (Figures S1b–S1d).

**Figure 4:**
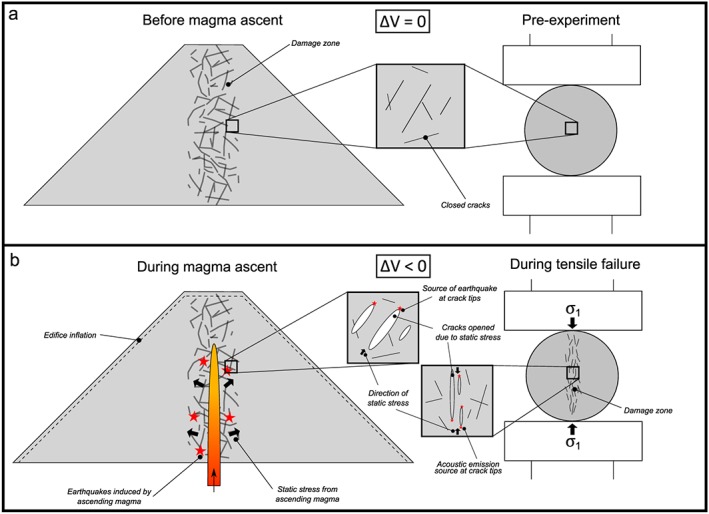
Two‐stage illustration of our seismic and experimental observations for the 1998 eruption of VdC: (a) Before magma ascent or experiment initiation and (b) during magma ascent or sample tensile failure.

Preeruptive velocity changes at other volcanoes have been linked to changes in stress in the volcano edifice. These changes may be induced by the expansion of pore spaces or cavities due to fluid saturation [*Grêt et al.*, 2006; *Sens‐Schönfelder and Wegler*, 2006], stress changes imposed by passing seismic waves [*Battaglia et al.*, 2012; *Hotovec‐Ellis et al.*, 2014; *Lesage et al.*, 2014], changes in surface snow load [*Hotovec‐Ellis et al.*, 2014], or magma propagating through the host rock [*Wegler et al.*, 2006; *Brenguier et al.*, 2008; *Hotovec‐Ellis et al.*, 2015]. Since the region had entered the dry season during our period of analysis, and no M5+ earthquakes were recorded within 800 km, we interpret our velocity change as a result of ascending magma. Our observations from Brazil tensile tests on samples from VdC lend support to this interpretation. Here failure of the sample is analogous to the failure of host rock during magma ascent prior to the eruption at VdC on 20 November (Figure 4). The measured porosities of our samples fall well within previously measured values on samples at VdC [*Lavallée et al.*, 2012] and are therefore representative of the materials making up the volcanic edifice [*Mueller et al.*, 2011]. The AE hit rate (Figures 3a, 3b, and S4) shows similar trends to those seen during the November 1998 swarm (Figure 2a). We observe a drop in the velocity of elastic waves traveling across the sample during the experiment (Figure 3c), replicating the velocity changes seen in ambient seismic noise (Figure 2c). This is indicative of fracture damage accumulation in the samples during loading [e.g., *Heap et al.*, 2010]. However, it must be noted that our experiments do not replicate the pressure and temperature conditions found at shallow depths in the volcanic edifice. Thus, the results can only be taken as a first‐order approximation of velocity changes associated with damage accumulation leading to tensile failure. A recent investigation concluded that higher confining (isostatic) pressures, analogous to greater depths in the crust, would dampen the reduction in elastic velocities due to crack opening [*Blake et al.*, 2013].

Clusters had previously been described prior to eruptions at VdC [*Varley et al.*, 2010] and other active volcanoes [e.g., *Hotovec‐Ellis et al.*, 2015]. Waveform correlation of all the earthquakes from October to December 1998 identified 504 clusters, with a vast majority occurring during the November swarm (Figure 2c). The number of short‐lived clusters indicate that multiple sources were repeatedly triggered during magma ascent (Figure 4b). Several source mechanisms have been inferred for clusters: “stick‐slip” failure on the margins of an ascending plug [e.g., *Iverson et al.*, 2006; *Kendrick et al.*, 2014; *Lamb et al.*, 2015], brittle failure of silicic magma on the conduit margins [e.g., *Neuberg et al.*, 2006; *Varley et al.*, 2010], and hydrothermal fluid motion [e.g., *Waite et al.*, 2008]. No lava plug was observed at the beginning of the eruption [*Zobin et al.*, 2002]; therefore, the stick‐slip mechanism is unlikely to have occurred. The low percentile of repeating events in the catalogue (7.3%) suggests the occurrence of a relatively low viscosity magma, no lava plug, and a high extrusion rate [*Thelen et al.*, 2011]; this agrees with observations during the eruption (e.g., the extrusion rate of 4.4 m^3^ s^−1^ and new lava flow [*Zobin et al.*, 2002]). We also detected AE clusters during our experiments to indicate repeated microcracking occurred prior to sample failure (Figures 3d–3f). We noticed an inverse correlation between the sample porosity and the number of AE clusters. This conforms with previous observations that increased porosity may act to inhibit dynamic fracture by crack arrest and/or by introducing a more heterogeneous stress field [*Kierfeld and Vinokur*, 2006; *Ramos et al.*, 2013]. We interpret the repeating events as the output of tensile, dynamic opening of new or existing fractures within the sample (Figure 4b). This suggests that tensile opening of cracks in the country rock due to static stress from propagating magma could also produce short‐lived clusters. Further work with rigorous testing of how sample heterogeneity may affect repetitive cracking and/or changes in velocity is required.

The velocity changes and the clusters at VdC during November 1998 may have been assisted by the presence of a “damage zone” surrounding the central volcanic conduit (Figure 4). The edifice at VdC is likely to be highly fractured and heterogeneous owing to the persistence of volcanic activity in recent centuries [*Breton Gonzalez et al.*, 2002]. Damage is observed at microscopic to macroscopic scales in the proximal deposits observed at VdC [e.g., *Lavallée et al.*, 2012]. This is supported by modeling at other dome‐forming volcanoes, for example, the existence of an intensely fractured damage zone surrounding the conduit can explain syn‐eruptive strain data recorded at Soufrière Hills volcano [*Young and Gottsmann*, 2015]. As demonstrated by our Brazil test experiments, static stress from the ascending magma can open preexisting or new fractures which reduces the velocity properties of the host rock (Figure 4b). Inflation and deflation of the volcanic edifice were observed around the 20 November 1998 eruption [*Ramírez‐Ruiz et al.*, 2002], illustrating the cycle of static stress caused by the ascent and eruption of magma through the edifice. In some cases, repeated activation can occur at the tips of the fractures as they open (Figure 4b). Tracking AE clusters during experiments on samples of different porosity demonstrates how the heterogeneity may also hinder further reactivation of earthquake sources. The recovery of seismic velocity and cessation of clusters at VdC can be explained by the release of static stress after the beginning of the eruption.

Here a combined field and laboratory investigation of seismic velocity changes associated with magma ascent has shown that velocity changes calculated via ambient seismic noise can be successfully used to assess eruption onset at Volcán de Colima. Furthermore, we have described evidence for dynamic crack propagation producing repetitive earthquakes that may have implications for future descriptions of preeruptive seismic activity. The study highlights the need for multidisciplinary investigative approaches for interpreting shallow processes associated with volcanic unrest.

## 5 Conclusions

A multidisciplinary approach was employed to investigate the seismic activity prior to the 20 November 1998 effusive eruption at Volcán de Colima, Mexico. Using seismic data recorded by the RESCO network of short‐period seismometers, we constructed a database of 17,893 earthquakes during this period. From this database, we identified 504 clusters using waveform correlation. Velocity changes during this period were measured using ambient seismic noise and indicate a ∼0.2% decrease prior to the eruption. We analyzed natural and artificial acoustic emissions recorded during Brazil tensile tests on andesite from Volcán de Colima. We observed a decrease in elastic wave velocity prior to sample failure, independent of sample porosity, which suggests that static stress due to ascending magma at Volcán de Colima was enough to induce velocity changes in the seismic data. We also observe a similarity in the pattern of clusters in the seismic and acoustic emission data, suggesting that tensile failure at crack tips is an additional viable source mechanism for clusters. This study highlights how using a multidisciplinary approach to understanding geophysical signals can help future interpretations of volcanic unrest and constrain eruption onset.

## Supporting information



Supporting Information S1
