# Supplementary material for: Seismic and experimental insights into eruption precursors at Volcán de Colima
Source: Geophys Res Lett. 2017 Jun 28;44(12):6092–100. doi: 10.1002/2017GL073350 (PMC6108408; doi:10.1002/2017GL073350)
Supplement: Supplementary file 1 — Supporting Information S1 [file GRL-44-6092-s001.pdf]

Supporting Information for "Seismic and experimental insights into eruption precursors at Volcán de Colima"

Oliver D. Lamb[1], Silvio De Angelis[1], Richard Wall[1], Anthony Lamur[1], Nick R. Varley[2], Gabriel Reyes-Dávila[3], Raul Arámbula-Mendoza[3], Adrian J. Hornby[1], Jackie E. Kendrick[1], Yan Lavallée[1]

Contents of this file

1. Figures S1 to S6
2. Table S1

Introduction

This supplementary information consists of Table S1 and Figures S1 to S6. Table S1 details the parameters used in MSNoise for calculating change in velocity using ambient

---

[1]Dept. of Earth, Ocean and Ecological Sciences, University of Liverpool, UK

[2]Facultad de Ciencias, Universidad de Colima, Mexico

[3]Centro Universitario de Estudios e Investigaciones Vulcanologicas, Universidad de Colima, Colima, Mexico

noise. Figure S1 shows the Real-time seismic amplitude (RSAM) of continuous seismic data collected at stations EZV3, EZV4 and EZV7, as well as seismic velocity calculations for different combinations of moving window stacks, frequency bands, and reference stack periods. Figure S2 shows the output of waveform correlation on seismic data using a threshold of 0.7. Output plots from MSNoise calculations are shown in figure S3. The set up for the Brazil test experiments is shown in figure S4. The results from all experiments carried out during this research are shown in figure S5. Figure S6 plots the seismic velocity calculations from acoustic emission pulses using the last pulse as a reference.

Table S1. Parameters used for calculating relative velocity change using the MSNoise ambient seismic noise package.

**MSNoise configuration**	
**Parameter**	**Value**
Analysis duration (s)	86400
Cross-correlation sampling rate (Hz)	20
Resampling method	*Decimate*
Pre-process filter limits (Hz)	0.01 - 8
Maximum lag (s)	120
Correlation duration	1800
Overlap (%)	50
Windsorizing	3
Stacking method	*Linear*
Reference begin	10/01/1998
Reference end	10/31/1998
DTT Lag	*Static*
Lag window (s)	10-40
DTT sides	*Both*
Minimum coherence	0.65
Maximum DTT value	0.08
Maximum error	0.1
**Filter configuration**	
**Parameter**	**Value**
Whiten filter (Hz)	0.5 - 1.1
MWCS filter (Hz)	0.5 - 1.1
MWCS window length (s)	12
MWCS window step (s)	4

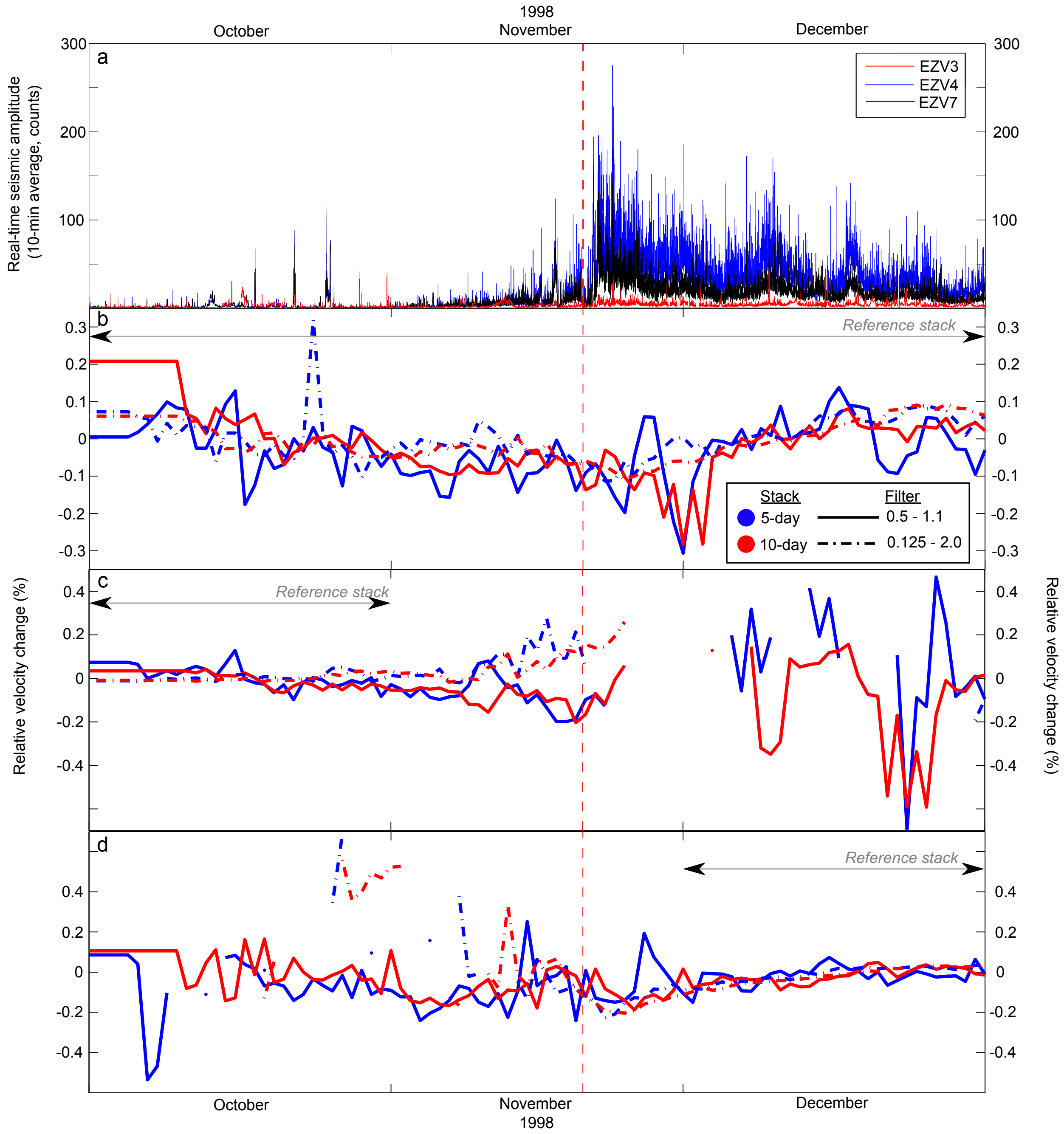

Figure S1. (a) 10-min RSAM values calculated from stations EZV3 (red line), EZV4 (blue line) and EZV7 (black line). The dashed red line represents the beginning of the eruption at Volcán de Colima on 20 November 1998.(b-d) Variations seismic velocity calculated from 5-day (blue) and 10-day (red) moving windows at frequency bands of 0.5-1.1 Hz (solid lines) and 0.125-2 Hz (dot-dashed lines). Each plot represents the seismic velocities calculated using reference stacks built from (b) the whole period, (c) October, and (d) December.

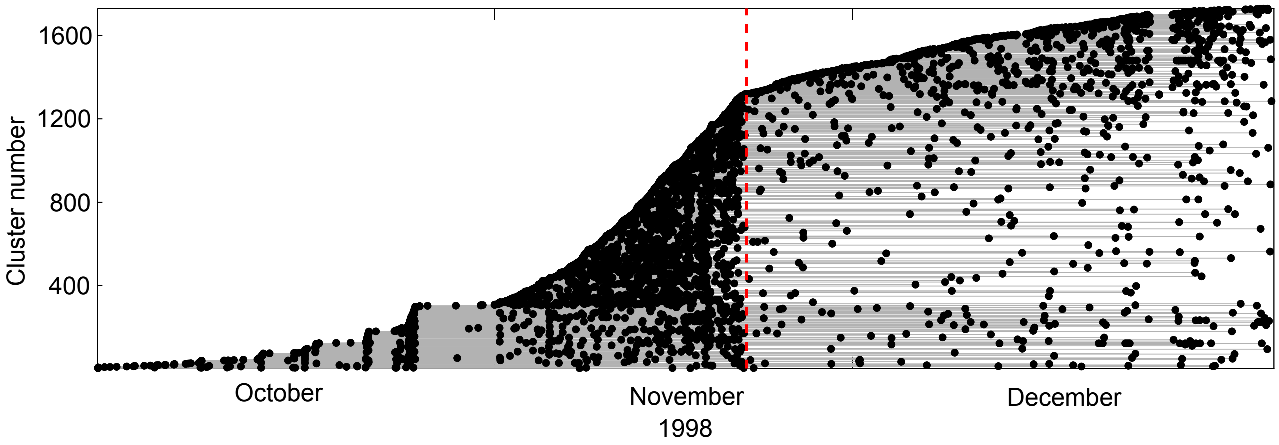

Figure S2. Catalogue of repeating earthquakes (black dots and grey lines) in our dataset from 1 October to 31 December 1998, using a coefficient threshold of 0.7. Each plotted point represents an individual earthquake, and each line joins groups of repeating events. The red dashed line represents the beginning of the effusive eruption at VdC on 20 November 1998.

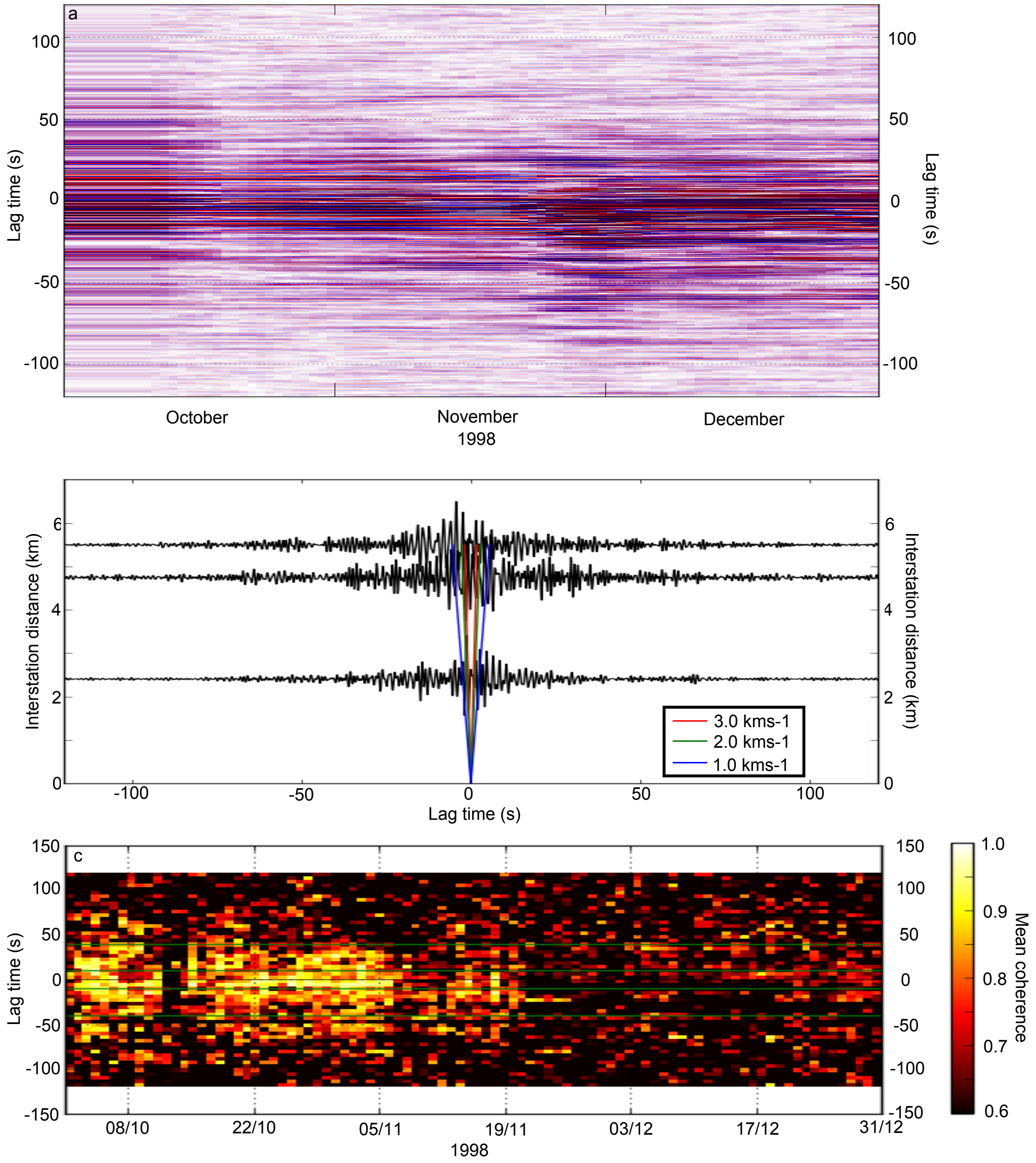

Figure S3. MSNoise output plots at 0.5 to 1.1 Hz frequency. (a) Interferogram of calculated cross-correlation functions for station pair EZV3-EZV7 (b) Reference stacks for each station pair plotted against interstation distance. Blue, red and green lines plot rays travelling at 1, 2 and 3 km s^{-1} respectively. (c) Mean coherence relative to the reference waveform for station pair EZV3-EZV7 for MWCS calculations. Selected time lags (10-40 s) for dt/t calculations are plotted as horizontal green lines.

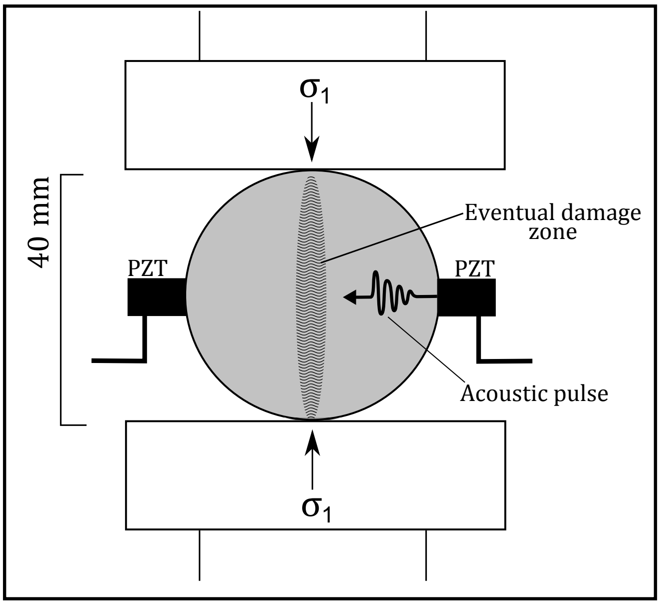

Figure S4. Illustration of the set-up for the Brazil test experiments conducted during this research.

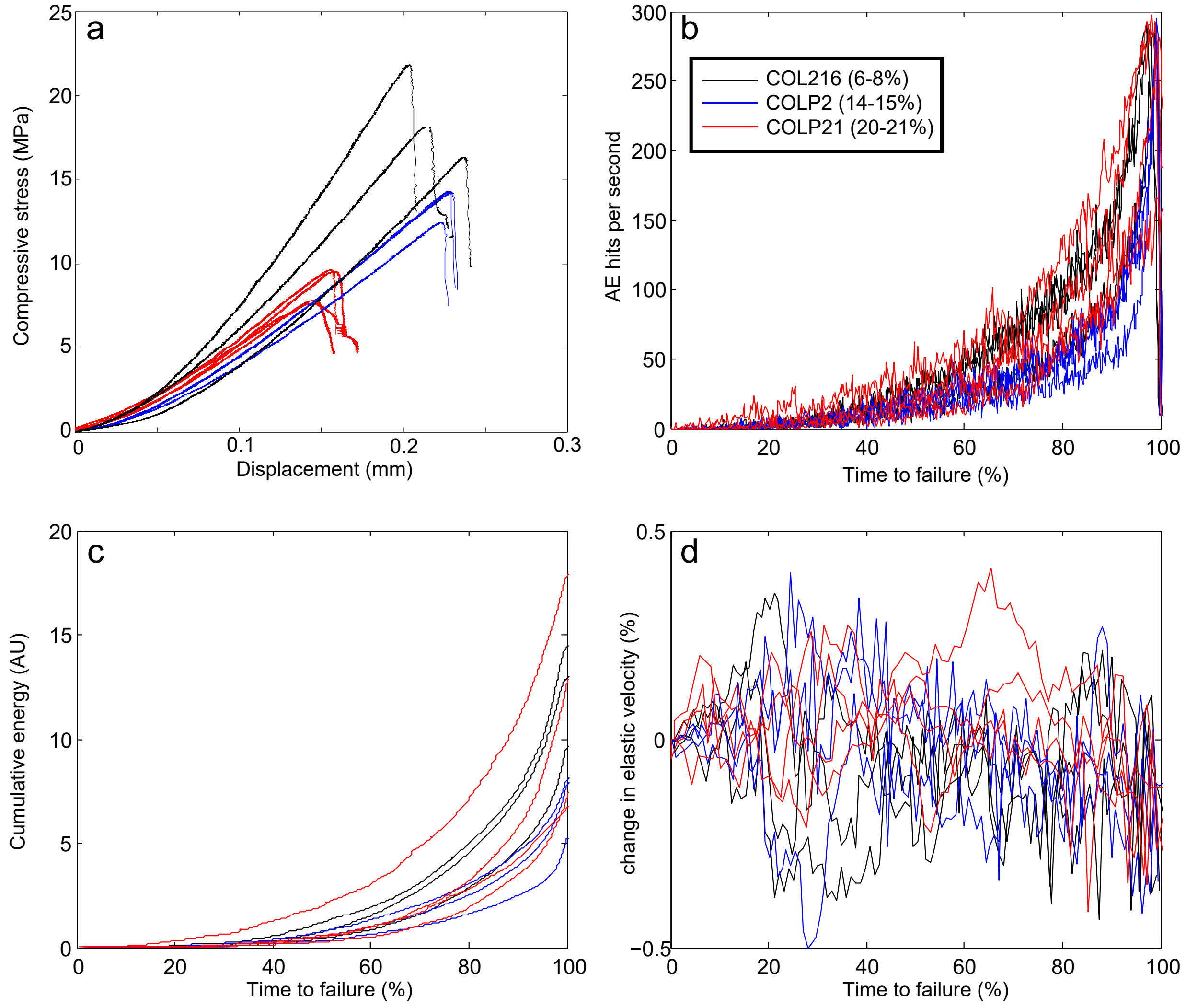

Figure S5. A compilation of results from all experiments carried out on COL216 (black), COLP2 (blue) and COLP21 (red) samples. (a) Displacement vs. stress recorded during the experiment. (b) Number of AEs recorded per second. (c) Cumulative energy of all AEs recorded during the experiment. (d) Change in velocity during the experiments.

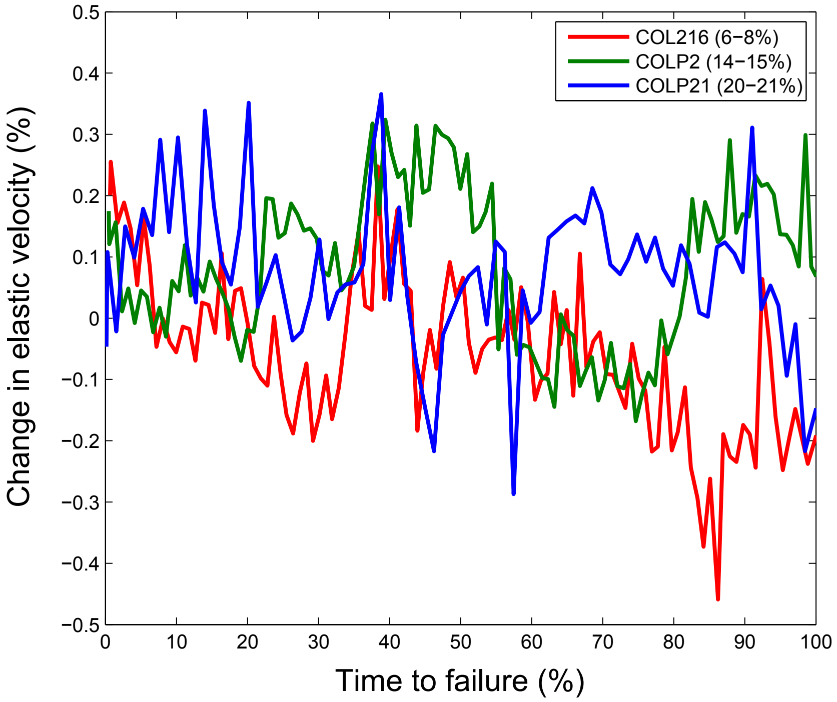

Figure S6. Change in velocity during the experiments, using the last pulse as the reference waveform.
